# Malaria-GENOMAP: a web-based tool for exploring genomic variation of malaria parasites

**DOI:** 10.1093/bioinformatics/btag016

**Published:** 2026-01-11

**Authors:** Joseph Thorpe, Nina Billows, Gabrielle C Ngwana-Joseph, Amy Ibrahim, Deborah Nolder, Colin J Sutherland, Thi Hong Ngoc Nguyen, Thi Huong Binh Nguyen, Quang Thieu Nguyen, Jamille G Dombrowski, Silvia Maria Di Santi, Claudio R F Marinho, Jody E Phelan, Tomasz Kurowski, Fady Mohareb, Susana Campino, Taane G. Clark

**Affiliations:** Faculty of Infectious and Tropical Diseases, London School of Hygiene & Tropical Medicine, Keppel St, London, WC1E 7HT, United Kingdom; Faculty of Infectious and Tropical Diseases, London School of Hygiene & Tropical Medicine, Keppel St, London, WC1E 7HT, United Kingdom; Faculty of Infectious and Tropical Diseases, London School of Hygiene & Tropical Medicine, Keppel St, London, WC1E 7HT, United Kingdom; Faculty of Infectious and Tropical Diseases, London School of Hygiene & Tropical Medicine, Keppel St, London, WC1E 7HT, United Kingdom; Faculty of Infectious and Tropical Diseases, London School of Hygiene & Tropical Medicine, Keppel St, London, WC1E 7HT, United Kingdom; UKHSA Malaria Reference Laboratory, London School of Hygiene & Tropical Medicine, Keppel St, London, WC1E 7HT, United Kingdom; Faculty of Infectious and Tropical Diseases, London School of Hygiene & Tropical Medicine, Keppel St, London, WC1E 7HT, United Kingdom; UKHSA Malaria Reference Laboratory, London School of Hygiene & Tropical Medicine, Keppel St, London, WC1E 7HT, United Kingdom; Molecular Biology Department, Parasitology and Entomology, Vietnam National Institute of Malariology, Hanoi, 100000, Vietnam; Molecular Biology Department, Parasitology and Entomology, Vietnam National Institute of Malariology, Hanoi, 100000, Vietnam; Molecular Biology Department, Parasitology and Entomology, Vietnam National Institute of Malariology, Hanoi, 100000, Vietnam; Department of Parasitology, Institute of Biomedical Sciences, University of São Paulo, Av. Prof. Lineu P , São Paulo, 05508-000, Brazil; Department of Medicine, University of São Paulo, Av. Dr. Arnaldo, 455 - Cerqueira César, São Paulo, 01246-903, Brazil; Department of Parasitology, Institute of Biomedical Sciences, University of São Paulo, Av. Prof. Lineu P , São Paulo, 05508-000, Brazil; Faculty of Infectious and Tropical Diseases, London School of Hygiene & Tropical Medicine, Keppel St, London, WC1E 7HT, United Kingdom; School of Water, Energy and Environment, Applied Bioinformatics, Cranfield University, Bedford, MK43 0AL, United Kingdom; School of Water, Energy and Environment, Applied Bioinformatics, Cranfield University, Bedford, MK43 0AL, United Kingdom; Faculty of Infectious and Tropical Diseases, London School of Hygiene & Tropical Medicine, Keppel St, London, WC1E 7HT, United Kingdom; Faculty of Infectious and Tropical Diseases, London School of Hygiene & Tropical Medicine, Keppel St, London, WC1E 7HT, United Kingdom; Faculty of Epidemiology and Population Health, London School of Hygiene and Tropical Medicine, Keppel St, London, WC1E 7HT, United Kingdom

## Abstract

**Motivation:**

Malaria, caused by *Plasmodium* parasites, imposes a significant public health burden. While *Plasmodium falciparum* remains the primary target of elimination strategies due to its high mortality rate, lesser-known species such as *P. malariae*, *P. vivax*, and *P. knowlesi* continue to contribute to substantial human morbidity. Genomic approaches, including whole-genome sequencing, offer powerful tools for understanding the biology, transmission, and emerging drug resistance of these neglected *Plasmodium* species. However, there is an urgent need for informatic tools to summarize and visualize the high-dimensional and complex genomic data generated.

**Results:**

We developed Malaria-GENOMAP, a user-friendly web-based tool, which integrates genomic variant data, such as allele frequencies, with geographical maps and chromosome-wide to gene views for in-depth exploration. The tool includes variation from *P. knowlesi* (*n* = 139), *P. malariae* (*n* = 158), *P. ovale curtisi* (*n* = 36), *P. ovale wallikeri* (*n* = 47), *P. simium* (*n* = 38), and *P. vivax* (*n* = 1359). It enables the investigation of population structure, geographic associations of mutations, and putative drug resistance markers, offering valuable insights for malaria control efforts.

**Availability and implementation:**

Malaria-GENOMAP is available online at https://genomics.lshtm.ac.uk/malaria-genomaps.

## 1 Introduction

Malaria, caused by *Plasmodium* parasites, led to 282 million cases and 610 000 deaths across 85 endemic countries in 2024 (World Health Organization 2025). While *P. falciparum* accounts for most global malaria mortality, neglected species like *P. vivax*, *P. simium*, *P. knowlesi*, *P. ovale curtisi*, *P. ovale wallikeri*, and *P. malariae* also cause significant disease. Among these, *P. vivax* is the most widespread outside Africa, causing over 14 million cases annually (World Health Organization 2025), while *P. knowlesi* is found in primates throughout Southeast Asia, and is the most common cause of human malaria in Malaysia (Singh *et al.* 2004, Turkiewicz *et al.* 2023). Diagnostic challenges often underestimate the prevalence of *P. malariae* and *P. ovale* spp parasites, which are commonly found in co-infections with *P. falciparum* (Tajebe *et al.* 2014). Furthermore, neglected malaria species contribute to elimination challenges through diverse biological traits, including chronic infections in *P. malariae*, hypnozoite-mediated relapses in *P. ovale* spp, zoonotic transmission, and the presence of asymptomatic carriers (Higgins *et al.* 2024).

Research on these species is limited, in part due to a lack of *in vitro* culture systems, but whole-genome sequencing (WGS) has advanced the understanding of parasite biology and epidemiology (Phelan *et al.* 2023). WGS technologies have expanded insights into genome diversity, drug resistance, and population structure for *P. falciparum* and neglected species. Tools like selective whole-genome amplification and molecular barcodes have enabled sequencing from low-parasitaemia samples and supported surveillance efforts (Benavente *et al.* 2021, Ibrahim *et al.* 2023, Ibrahim *et al.* 2024, Turkiewicz *et al.* 2023, Ngwana-Joseph *et al.* 2024). However, a gap remains in tools for analyzing and summarizing genomic data for neglected malaria species, as existing platforms focus predominantly on *P. falciparum* and typically require substantial bioinformatics expertise to explore genomic variation across geographic regions (e.g. https://apps.malariagen.net/apps/pf7/). Other resources that include genomic data from neglected *Plasmodium* species, such as PlasmoDB (https://plasmodb.org/plasmo/app), contain more limited population-level data and are primarily oriented towards genome annotation rather than population structure, and are largely targeted at malaria biology specialists. Overall, there is a lack of interactive, geography-aware tools that enable non-specialist users to explore population structure, allele frequencies, and putative drug resistance markers across the various neglected *Plasmodium* species.

To address this, we present Malaria-GENOMAP, a web-based tool aggregating data from >1700 *Plasmodium* genomes, including SNPs in drug resistance genes and allele frequencies across endemic regions. Designed for neglected species, the tool supports diagnostics, drug development, and surveillance activities.

## 2 Methods

### 2.1 Malaria-GENOMAP database

**Figure 1:**
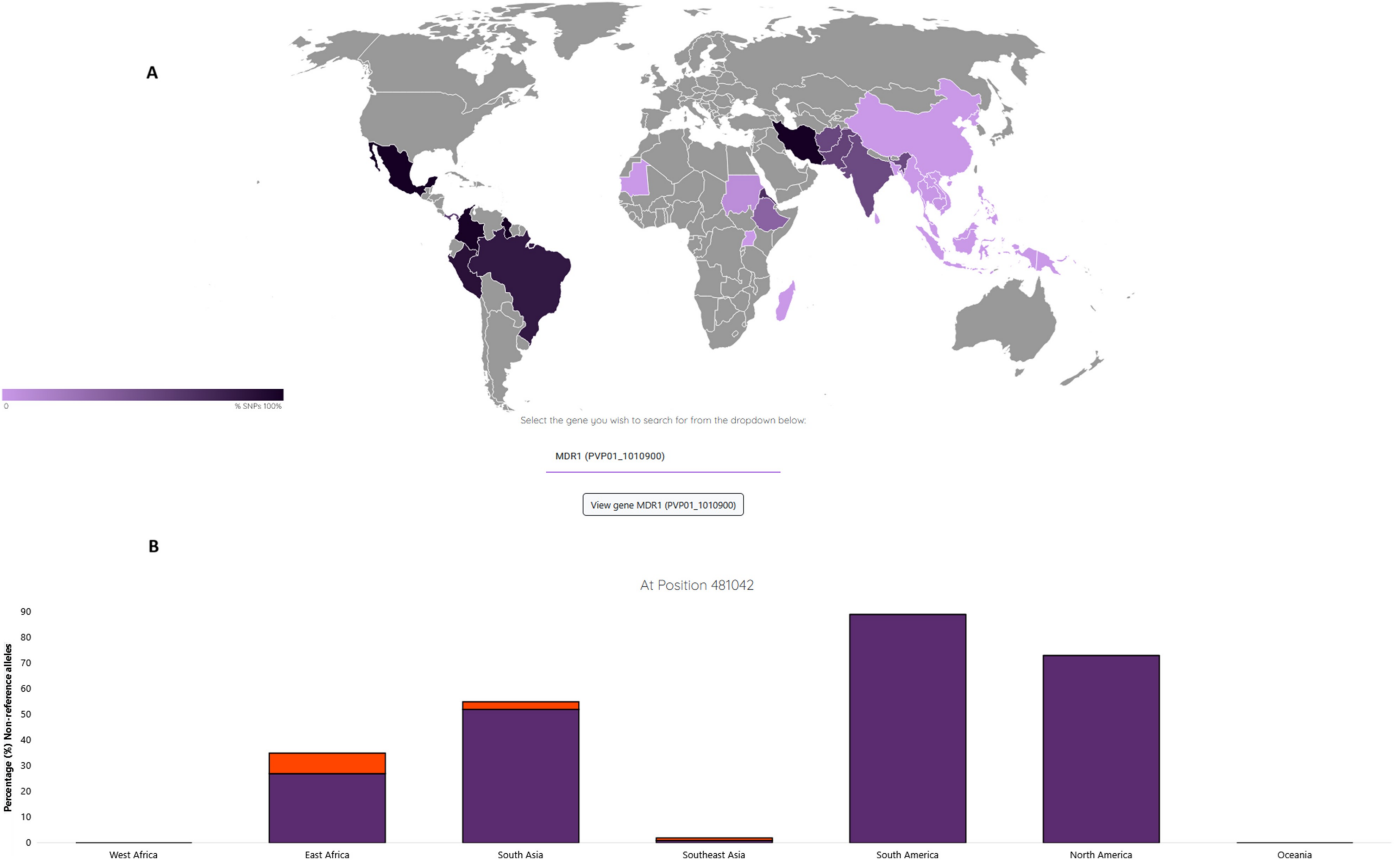
Malaria-GENOMAP Screenshots. (A) Countries represented with *P. vivax* data; (B) Pvmdr1 gene (position 481042 T -> C; C698S) shows population differentiation between South America and Southeast Asia *P. vivax* populations (%).

The dataset includes clinical isolates of *Plasmodium* from single-species infections, encompassing: *P. malariae* (*n* = 158) (Ibrahim *et al.* 2024), *P. vivax* (*n* = 1359) (Ibrahim *et al.* 2023, Ngwana-Joseph *et al.* 2024), *P. simium* (*n* = 38) (Manko *et al.* 2025), *P. ovale* spp (*wallikeri n* = 47; *curtisi n* = 36) (Higgins *et al.* 2024), and *P. knowlesi* (*n* = 139) (Turkiewicz *et al.* 2023) (Table 1). To identify SNPs and insertions or deletions (indels), raw FASTQ files were mapped to reference genomes using bwa-mem software (v0.7.12) (Li and Durbin 2009). Variants were called with GATK’s HaplotypeCaller (v4.1.4.1) (McKenna *et al.* 2010), using the -ERC GVCF option to generate a combined VCF file for all isolates. This file was filtered to include only SNPs in core genomes and further refined by excluding SNPs with a negative Variant Quality Score Log-Odds. Where training SNP datasets were unavailable, SNPs were filtered using the GATK VariantFiltration function. Annotated species-specific VCF files were indexed with existing GFF3 annotation data. Associated metadata, such as collection year and location, were compiled into a TSV file. Finally, SNP and metadata were merged into a MySQL database for each species, enabling efficient user-driven queries (Fig. 1, available as supplementary data at *Bioinformatics* online).

**Table 1:** Malaria-GENOMAP isolate database.

Species	Genome size	No. SNPs	No. isolates	No. countries
*P. knowlesi*	24.4 Mbp	2 051 729	158	2
*P. malariae*	29.6 Mbp	221 656	139	26
*P. ovale curtisi*	36.0 Mbp	268 850	36	16
*P. ovale wallikeri*	34.3 Mbp	216 320	47	22
*P. simium*	21.7 Mbp	123 902	38	1
*P. vivax*	24.2 Mbp	535 146	1359	29

### 2.2 Malaria-GENOMAP framework

The database is visualized through an intuitive interface built with the Angular framework and amCharts (2024) (https://www.amcharts.com/javascript-charts). Backend operations, powered by Node.js, retrieve and display JSON-formatted data from the MySQL database with minimal query response times. Tables are indexed for rapid searches, and users can interact with three main views:

1. Country: Displays data availability and annotated genomic variations (e.g. amino acid changes) for specific countries.
2. Gene: Allows searches by locus or variant, showing global frequencies of occurrence by country, with outputs in tabular format.
3. Alignment: Integrates IGV tools to examine variants within genomic regions, providing quality metrics and expandable annotation tracks.

The platform is accessible via https://genomics.lshtm.ac.uk/malaria-genomaps/#/, offering dynamic maps, graphs, and tables to support diverse research and surveillance needs (Fig. 2, available as supplementary data at *Bioinformatics* online).

## 3 Results

To highlight the functionality of Malaria-GENOMAP, we show some examples of its use. Markers of population differentiation may be useful for molecular barcoding and can be driven by drug resistance or mosquito vector diversity. For *P. vivax*, previous work has found selective sweeps proximal to *pvmdr1*, a putative marker for chloroquine resistance (Ngwana-Joseph *et al.* 2024). Using Malaria-GENOMAP, we highlight a nonsynonymous SNP leading to amino acid substitution 698S > 698G in *pvmdr1* (445/1294, 34.4%), which is near fixed in South American population (264/297, 88.9%) compared to Southeast Asia (7/550, 1.3%) (Fig. 1), consistent with previous studies (Ibrahim *et al.* 2023). Similarly, the *PVP01_1313400* K841N mutation is highly frequent in East Africa (Ethiopia 135/137, 98.5%; Eritrea 11/13, 84.6%; Uganda 3/5, 60%), but absent in Asia and South America (0/297, 0%) (Fig. 3, available as supplementary data at *Bioinformatics* online) (Benavente *et al.* 2021). Whilst the *PvP47* and *PvP48/45* genes are linked to the mosquito vector, with *P47* K27E mutation (*P48/45* R418K) found near fixed in South America (278/297, 93.6%) but absent elsewhere (0/1062, 0%) (Fig. 3, available as supplementary data at *Bioinformatics* online) (Benavente *et al.* 2021).

The *pfdhfr* gene in *P. falciparum* has been linked to pyrimethamine resistance (Turkiewicz *et al.* 2020), with five amino acid variants within the *pmdhfr* (A15S, S49R, F57L, R58S and N114S) in *P. malariae* aligning closely with mutations linked to drug susceptibility in *pfdhfr* (Ibrahim *et al.* 2024). The *pmdhfr* N114S mutation is prevalent globally (114/151, 75.5%), with high frequencies in mid-Africa (27/33, 81.8%), West Africa (23/29, 79.3%), and South America (6/6, 100%), with high-quality allele calls checked using the IGV view (Fig. 4, available as supplementary data at *Bioinformatics* online). This activity demonstrates the potential for Malaria-GENOMAP to discover any future SNP mutations in neighbouring regions, as well as identify new SNPs that can be linked to drug resistance.

## 4 Discussion

Malaria remains a critical public health issue, with genomics research largely concentrated on *P. falciparum*, the deadliest of the *Plasmodium* species, evidenced by existing tools that focus primarily on this species [https://apps.malariagen.net/apps/pf7/; Pf-HaploAtlas (Lee *et al.* 2024)]. This focus often leaves other *Plasmodium* species underrepresented, which presents challenges for achieving WHO’s malaria elimination targets (World Health Organization 2025). With advancements in sequencing technology now enabling data generation from infections with low parasitaemia, particularly for neglected malaria species, our work aims to fill this gap by providing and visualizing comprehensive genomic data. Our web-based tool, Malaria-GENOMAP, allows non-specialist users to interactively explore population structure, allele frequencies, and putative drug-resistance markers across multiple neglected *Plasmodium* species. These insights shed light on genetic variations that are essential for understanding infection control and population diversity (Phelan *et al.* 2023, Ibrahim *et al.* 2024). The tool aggregates and visualizes SNP data from >1700 high-quality samples, highlighting mutations linked to drug resistance and their regional specificity. This information supports the development of molecular barcodes, which can track transmission patterns, and informs the design of improved diagnostics and vaccines (Preston *et al.* 2014, Benavente *et al.* 2020). Looking forward, Malaria-GENOMAP is designed to incorporate additional data from neglected malaria species, with the potential to expand to include new functionalities such as predictive modelling for drug resistance mutations. For instance, advanced machine learning algorithms could be integrated to anticipate resistance patterns before they emerge, informing more targeted interventions.

In summary, Malaria-GENOMAP is positioned to play a role in advancing malaria research by illuminating genomic variation across neglected *Plasmodium* species. This tool provides invaluable resources for research and surveillance efforts focused on eradicating malaria and reducing its global health burden.

## Supplementary Material

btag016_Supplementary_Data

## Data Availability

The data underlying this article will be shared on reasonable request to the corresponding author.

## References

[btag016-B1] amCharts: JavaScript Charts. amCharts. Accessed 10 December 2024.

[btag016-B2] Benavente ED , Campos M, Phelan J et al A molecular barcode to inform the geographical origin and transmission dynamics of plasmodium vivax malaria. PLoS Genet 2020;16:e1008576. 10.1371/journal.pgen.1008576 32053607 PMC7043780

[btag016-B3] Benavente ED , Manko E, Phelan J et al Distinctive genetic structure and selection patterns in Plasmodium vivax from South Asia and East Africa. Nat Commun 2021;12:3160. 34039976 10.1038/s41467-021-23422-3 PMC8154914

[btag016-B4] Higgins M , Manko E, Ward D et al New reference genomes to distinguish the sympatric malaria parasites, Plasmodium ovale curtisi and Plasmodium ovale wallikeri. Sci Rep 2024;14:3843. 38360879 10.1038/s41598-024-54382-5 PMC10869833

[btag016-B5] Ibrahim A , Mohring F, Manko E et al Whole genome sequencing of Plasmodium malariae identifies continental segregation and mutations associated with reduced pyrimethamine susceptibility. Nat Commun 2024;15:10779. 39738025 10.1038/s41467-024-55102-3 PMC11685946

[btag016-B6] Ibrahim A , Manko E, Dombrowski JG et al Population-based genomic study of *Plasmodium vivax* malaria in seven Brazilian states and across South America. Lancet Reg Health Am 2023;18:100420. 36844008 10.1016/j.lana.2022.100420 PMC9950661

[btag016-B7] Lee C , Ünlü ES, White NFD et al Pf-HaploAtlas: an interactive web app for spatiotemporal analysis of *Plasmodium falciparum* genes. Bioinformatics 2024;40:btae673. 39565917 10.1093/bioinformatics/btae673 PMC11588202

[btag016-B8] Li H , Durbin R. Fast and accurate short read alignment with Burrows–Wheeler transform. Bioinformatics 2009;25:1754–60. 19451168 10.1093/bioinformatics/btp324 PMC2705234

[btag016-B9] McKenna A , Hanna M, Banks E et al The genome analysis toolkit: a MapReduce framework for analyzing next-generation DNA sequencing data. Genome Res. 2010;20:1297–303. 20644199 10.1101/gr.107524.110 PMC2928508

[btag016-B10] Manko E , Billows N, Higgins M et al Novel zoonotic cases of *plasmodium simium* from Sao Paulo with the first reference genome of the Brazilian strain. Sci Rep 2025;15:43703. 41387489 10.1038/s41598-025-27554-0 PMC12700903

[btag016-B11] Ngwana-Joseph GC , Phelan JE, Manko E et al Genomic analysis of global Plasmodium vivax populations reveals insights into the evolution of drug resistance. Nat Commun 2024;15:10771. 39738010 10.1038/s41467-024-54964-x PMC11685768

[btag016-B12] Phelan JE , Turkiewicz A, Manko E et al Rapid profiling of plasmodium parasites from genome sequences to assist malaria control. Genome Med 2023;15:96. 37950308 10.1186/s13073-023-01247-7 PMC10636944

[btag016-B13] Preston MD , Campino S, Assefa SA et al A barcode of organellar genome polymorphisms identifies the geographic origin of Plasmodium falciparum strains. Nat Commun 2014;5:4052. 24923250 10.1038/ncomms5052 PMC4082634

[btag016-B14] Singh B , Kim Sung L, Matusop A et al A large focus of naturally acquired Plasmodium knowlesi infections in human beings. Lancet 2004;363:1017–24. 15051281 10.1016/S0140-6736(04)15836-4

[btag016-B15] Tajebe A , Magoma G, Aemero M et al Detection of mixed infection level of Plasmodium falciparum and Plasmodium vivax by SYBR green I-based real-time PCR in North Gondar, North-west Ethiopia. Malar J 2014;13:411. 25326079 10.1186/1475-2875-13-411 PMC4210478

[btag016-B16] Turkiewicz A , Manko E, Sutherland CJ et al Genetic diversity of the Plasmodium falciparum GTP-cyclohydrolase 1, dihydrofolate reductase and dihydropteroate synthetase genes reveals new insights into sulfadoxine-pyrimethamine antimalarial drug resistance. PLoS Genet 2020;16:e1009268. 33382691 10.1371/journal.pgen.1009268 PMC7774857

[btag016-B17] Turkiewicz A , Manko E, Oresegun DR et al Population genetic analysis of Plasmodium knowlesi reveals differential selection and exchange events between Borneo and peninsular sub-populations. Sci Rep 2023;13:2142. 36750737 10.1038/s41598-023-29368-4 PMC9905552

[btag016-B18] World Health Organization. World Malaria Report 2025. Geneva: World Health Organization, 2025.

