# Supplementary material for: Malaria-GENOMAP: a web-based tool for exploring genomic variation of malaria parasites
Source: Bioinformatics. 2026 Jan 11;42(2):btag016. doi: 10.1093/bioinformatics/btag016 (PMC12881832; doi:10.1093/bioinformatics/btag016)

Malaria genomaps supplementary material

S1 Pipeline

1. Pipeline from raw Fastq to filtered VCF

B) Back end and front end of the server


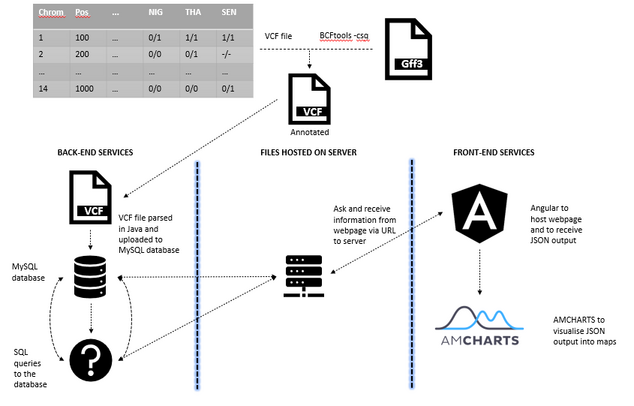


Overview of the structure of the project. Genetic variants from the GT field of a VCF file were annotated with the annotation file and BCFtools command -csq. The now annotated file is then parsed using a custom script in Java, and populated into a MySQL database, where queries depending on the URL can relay specific data to the user. These files are hosted on a private server and the output from the queries are visualised on a webpage created with Angular and in map, chart and table form using amCharts.


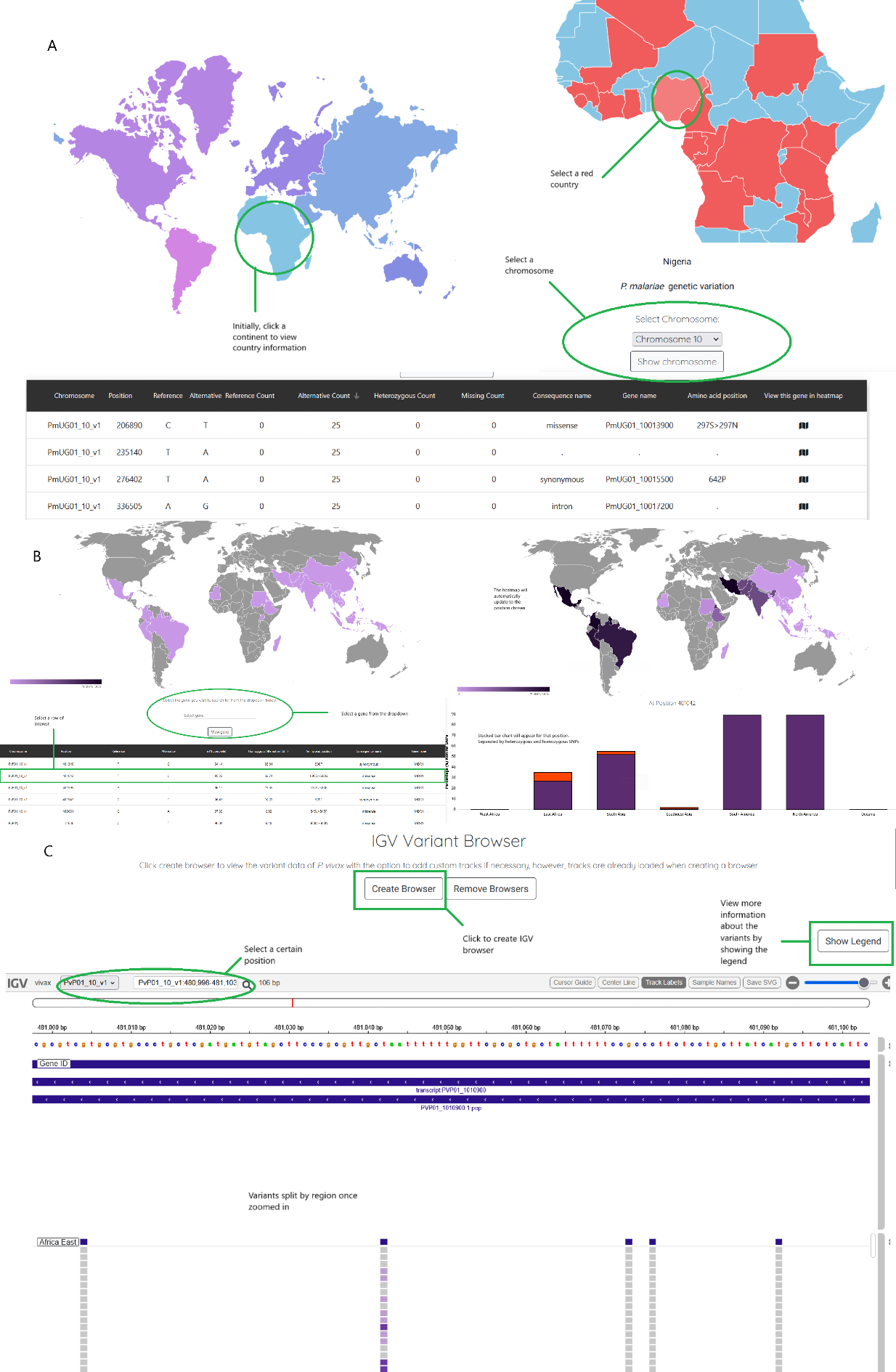
S2
(A) Chromosome View
(B) Heatmap View
(C) IGV View

S3

**Population differentiation in *P. vivax***

**(A)** *PVP01_1313400* (chr 13, position 610100 C->A; K841N) which is highly frequent in Ethiopia (135/137) and Eritrea (11/13), but absent in Asia and South America (0/297)

**(B)** *P47* gene (chr 12, position 323612 G->A; K27E) linked to the mosquito vector found in South America (278/297) but absent elsewhere (0/1062)


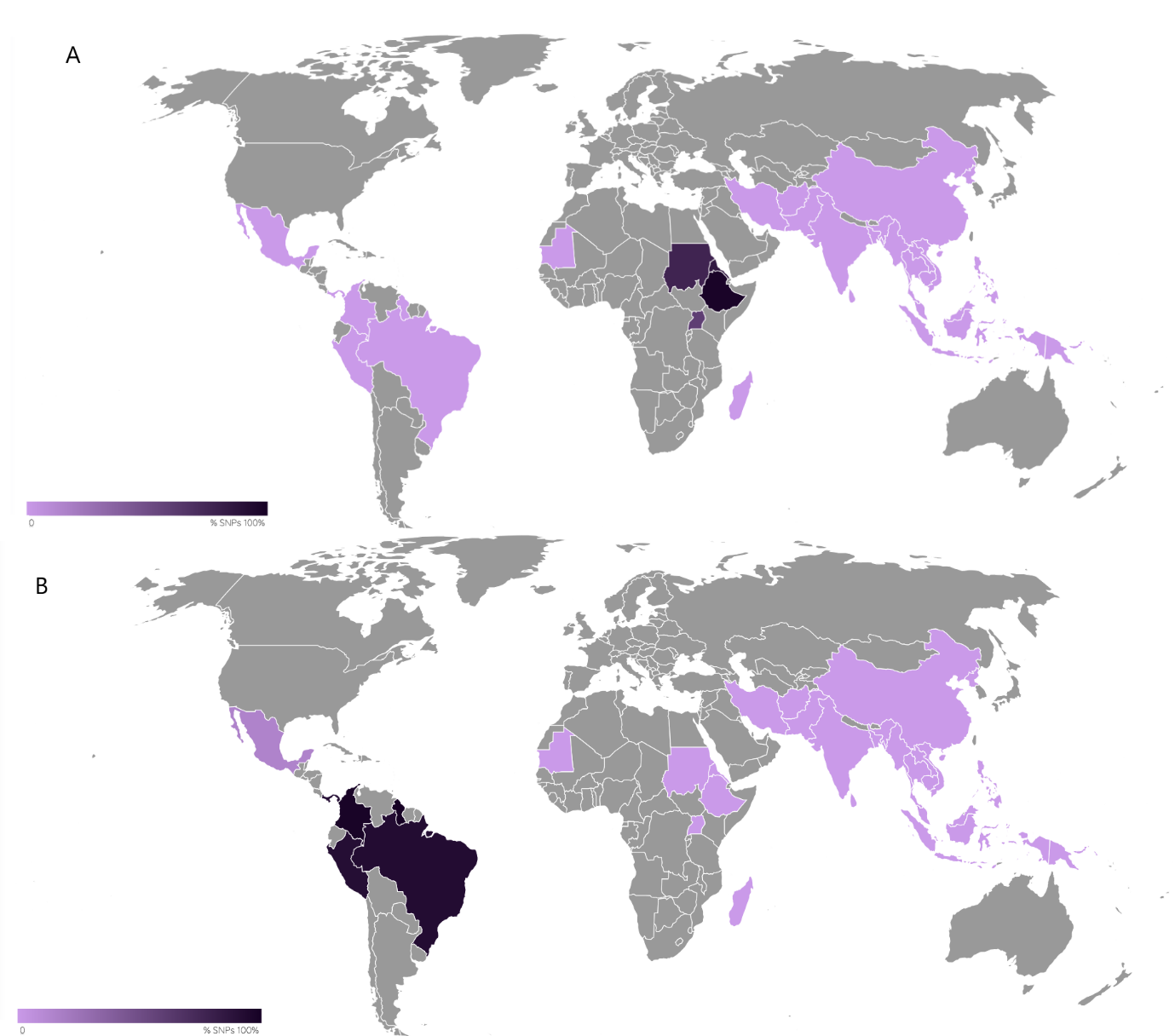


S4 **Pyrimethamine resistance drug resistance orthologue for *Pmdhfr* N114S (1292193 A -> G)**

1. **The heatmap view for this SNP**
2. **The same view, but the bar chart section of the same SNP.**


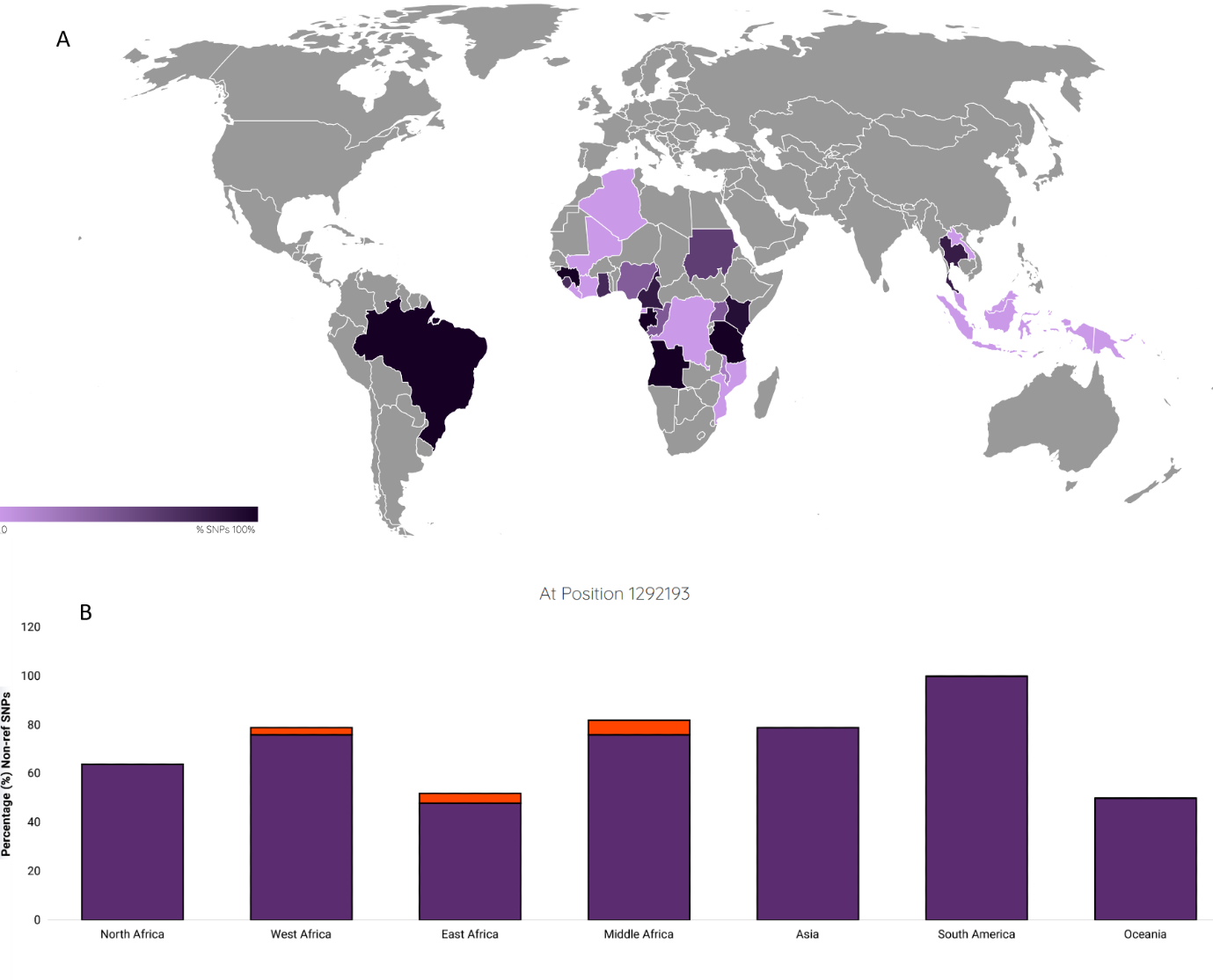

Supplement: btag016_Supplementary_Data [file btag016_supplementary_data.docx]
